# Associations of General and Central Obesity with Cardiovascular Risk by Menopausal and MASLD Status

**DOI:** 10.3390/biomedicines14091985

**Published:** 2026-09-03

**Authors:** Dong Yun Kim, Kyu-Na Lee, Kyung-Do Han, Beom Kyung Kim

**Affiliations:** 1 Department of Internal Medicine, Yonsei University College of Medicine, 50-1 Yonsei-ro, Seodaemun-gu, Seoul 03722, Republic of Korea; 2 Institute of Gastroenterology, Yonsei University College of Medicine, 50-1 Yonsei-ro, Seodaemun-gu, Seoul 03722, Republic of Korea; 3 Yonsei Liver Center, Severance Hospital, Yonsei University Health System, 50-1 Yonsei-ro, Seodaemun-gu, Seoul 03722, Republic of Korea; 4 Department of Public Health, The Catholic University of Korea, 222 Banpo-daero, Seocho-gu, Seoul 06591, Republic of Korea; 5 Department of Statistics and Actuarial Science, Soongsil University, 369 Sangdo-ro, Dongjak-gu, Seoul 06978, Republic of Korea

**Keywords:** menopause, metabolic dysfunction-associated steatotic liver disease, cardiovascular disease, central obesity, body mass index

## Abstract

**Background/Objectives:** Metabolic dysfunction-associated steatotic liver disease (MASLD) prevalence increases during the menopausal transition, yet its interaction with obesity phenotypes in influencing cardiovascular disease (CVD) risk remains unclear. We examined how MASLD and obesity types are associated with CVD risk in pre- and postmenopausal women. **Methods:** This nationwide cohort analyzed 1,959,367 Korean women aged ≥40 years (848,672 premenopausal, 1,110,695 postmenopausal) followed for a median of 12.3 years. MASLD was defined by the fatty liver index plus ≥1 cardiometabolic risk factor and alcohol intake <20 g/day. General obesity was defined as body mass index ≥25 kg/m^2^ and central obesity as waist circumference ≥85 cm. **Results:** During follow-up, 106,792 women (5.45%) developed CVD. MASLD was associated with higher CVD risk regardless of menopausal status, though the association was numerically smaller in postmenopausal women. Among women without MASLD, general obesity was associated with risk before menopause (adjusted hazard ratio [aHR] 1.10, 95% confidence interval [CI] 1.04–1.17; central obesity, aHR 1.06, 95% CI 0.96–1.18), whereas after menopause central obesity was associated with risk (aHR 1.08, 95% CI 1.05–1.10). In postmenopausal women with MASLD, the estimate for general obesity was inverse (aHR 0.95) while central obesity remained positively associated (aHR 1.05); in an exploratory sensitivity analysis using an anthropometry-free metabolic proxy (the triglyceride–glucose index), the inverse estimate was not reproduced (aHR 1.06). **Conclusions:** MASLD was associated with elevated CVD risk across menopausal stages. Associations with obesity phenotype varied by menopausal status: general obesity was associated with risk in premenopausal women without MASLD, whereas after menopause the association was more consistent for central obesity. These are observational contrasts between groups that also differ substantially in age and should not be interpreted causally. They may nonetheless inform prevention tailored to menopausal stage, MASLD status, and obesity phenotype.

## 1. Introduction

Cardiovascular disease (CVD) remains the leading cause of mortality in women worldwide, and risk profiles differ significantly between the pre- and postmenopausal periods [1]. Among the various cardiometabolic risk factors (CMRFs), obesity and metabolic dysfunction-associated steatotic liver disease (MASLD) have emerged as major contributors to cardiovascular outcomes [2,3,4,5,6]. Chronic low-grade inflammation is common to all three conditions. Systemic inflammatory burden, quantified by indices such as the systemic immune-inflammation index, has been associated with incident cardiovascular disease including myocardial infarction and ischemic stroke [7]; adipose tissue expansion and dysfunction sustain this state through altered adipokine and cytokine secretion, and reducing adiposity attenuates the accompanying metabolic derangements [8]; and in the liver, inflammation rather than steatosis drives progression to steatohepatitis [9]. Obesity, MASLD, and CVD therefore converge on a shared inflammatory axis, providing the rationale for examining them jointly. The interplay between these conditions and the hormonal shifts that occur during menopause has not been fully elucidated, particularly in the context of sex-specific pathophysiological mechanisms [10,11].

General obesity, typically assessed by body mass index (BMI), is a well-established risk factor for both MASLD and CVD. While it reflects overall adiposity, it does not adequately differentiate fat distribution or capture metabolic risk [12]. Reflecting these limitations, indices combining anthropometric with biochemical measurements have attracted growing interest; the visceral adiposity index, derived from body mass index, waist circumference, triglycerides, and high-density lipoprotein cholesterol, has been related to insulin resistance, dysglycaemia, steatotic liver disease, and metabolic syndrome, and evaluated as a predictor of cardiovascular risk [13]. In contrast, central obesity—measured by waist circumference or the waist-to-height ratio—represents excess visceral fat accumulation around the abdominal organs, which has greater metabolic and cardiovascular implications. Visceral adipose tissue is more metabolically active and pro-inflammatory than subcutaneous fat, secreting higher levels of adipokines (e.g., resistin, leptin) and pro-inflammatory cytokines (e.g., IL-6, TNF-α), while reducing protective adiponectin levels. Recent evidence indicates that menopause, independent of chronological aging, drives adverse changes in body composition. This transition is marked by a shift toward visceral adiposity, now recognized as a key mediator of metabolic dysfunction, systemic inflammation, and endothelial dysfunction, thereby accelerating atherogenesis [14,15]. In parallel, molecular profiling has begun to define signatures that distinguish progressive from non-progressive disease, indicating that steatosis, inflammation, and fibrogenesis represent biologically distinct states rather than a single continuum [16]. Given the shift toward central adiposity, insulin resistance, and chronic inflammation after menopause, women become further predisposed to ectopic fat deposition and vascular dysfunction [17]. Therefore, further research is required to develop effective preventive strategies to reduce CVD risk in pre- and postmenopausal women [17].

MASLD, recently redefined to emphasize its metabolic underpinnings [18], is increasingly recognized not merely as a hepatic condition but as a systemic disease strongly associated with atherosclerosis, subclinical cardiac dysfunction, and major CVD. However, data on sex-specific and menopause-stage-specific interactions remain limited. In particular, there is a lack of large-scale population-based evidence addressing how these interrelationships evolve across the menopausal transition. Therefore, it is clinically crucial to determine whether the cardiovascular risk conferred by MASLD differs according to menopausal stage and obesity phenotype (general vs. central). Clarifying these interactions could refine risk stratification and guide personalized interventions, particularly with the advent of novel MASLD pharmacotherapies [19,20]. This study therefore aimed to investigate the distinct and combined associations of general and central obesity with cardiovascular risk according to menopausal and MASLD status, using a large-scale nationwide South Korean cohort, with the goal of refining cardiovascular risk stratification and informing targeted prevention strategies in women.

## 2. Materials and Methods

### 2.1. Database

Data from the Korean National Health Insurance Service (NHIS) were used for this analysis. The NHIS is a mandatory and comprehensive health insurance system covering 97% of the South Korean population. It provides periodic health examinations—including anthropometric measurements, self-administered lifestyle questionnaires, and laboratory assays—at least every two years to nearly all South Koreans. The NHIS data encompass demographic information, health check-up records, and diagnoses and treatments coded according to the International Statistical Classification of Diseases and Related Health Problems, Tenth Revision (ICD-10) [21].

The National Cancer Screening Program is part of the NHIS health screening initiative. Within its questionnaire, there is a section exclusively for women that includes questions regarding current menstrual status. Participants can select one of three responses: “I still have regular periods,” “I have undergone a hysterectomy,” or “I am menopausal.” If the response is “I am menopausal,” an additional question requests the respondent to report the age at menopause. The survey also includes questions about age at menarche and, if applicable, the duration of hormone replacement therapy (HRT), categorized as 0, <2, 2–4, or ≥5 years.

### 2.2. Study Population

**Figure 1:**
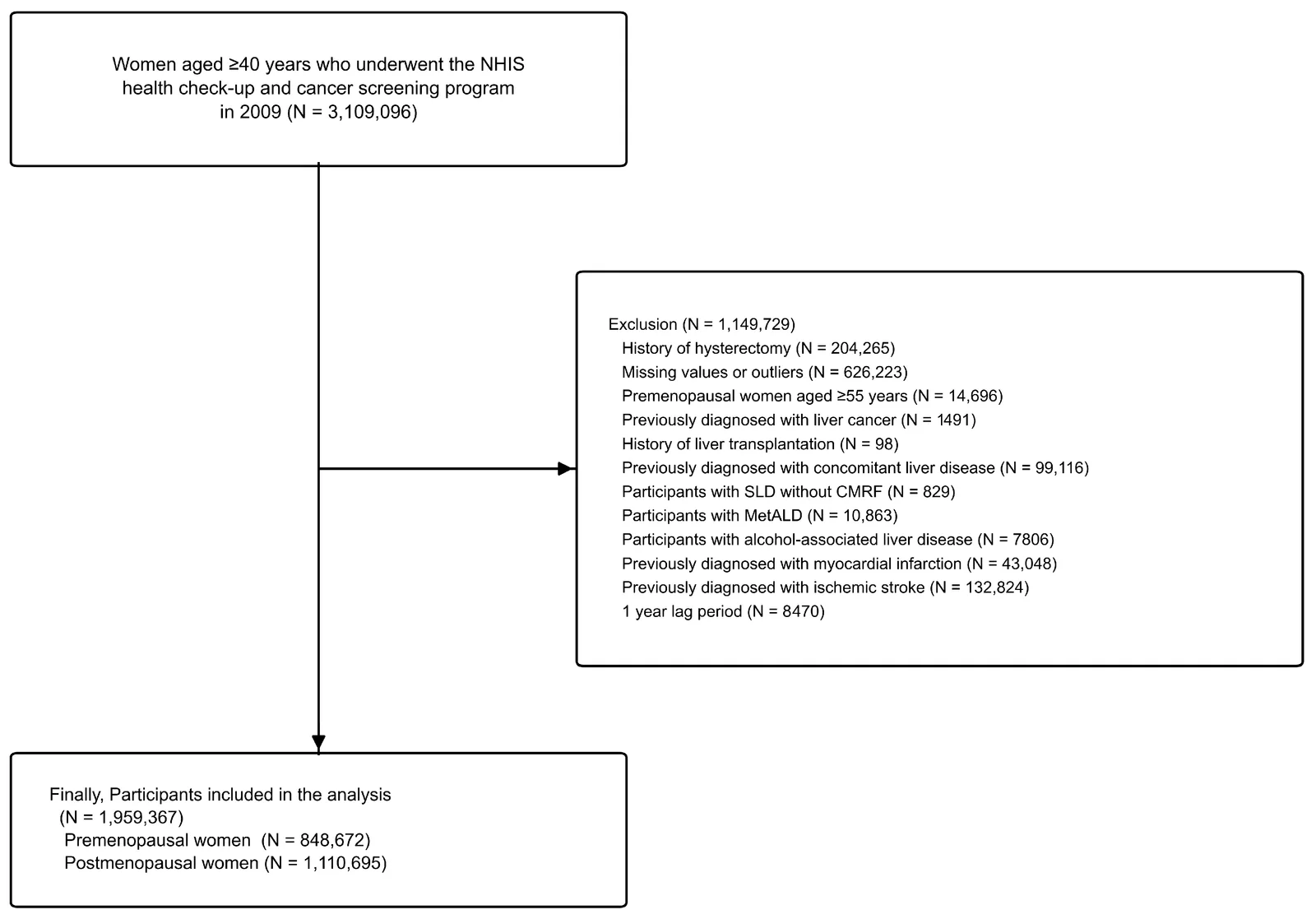
Flowchart of the participant recruitment process.

Of the participants who underwent health screenings in 2009, women aged ≥40 years were initially extracted from the NHIS database (n = 3,109,096). The exclusion criteria were as follows: history of hysterectomy, missing questionnaire values or outliers (e.g., menarche before age 5 or after age 30), premenopausal women aged ≥55 years, previous diagnosis of liver cancer, history of liver transplantation, diagnosis of concomitant liver disease, steatotic liver disease (SLD) without CMRFs, metabolic dysfunction-associated steatotic liver disease with increased alcohol intake (MetALD), alcohol-associated liver disease (ALD), previous diagnosis of myocardial infarction or ischemic stroke, and a lag period of less than one year (Figure 1). Prior peripheral arterial events—critical limb ischemia, amputation for critical limb ischemia, and mesenteric ischemia—were not applied as exclusion criteria, since they did not form part of the study endpoint (Section 2.4). Table S1 shows the ICD-10 codes used to define concomitant liver disease, ALD, alcohol abuse, and alcohol misuse.

This study adhered to the ethical principles of the 2013 Declaration of Helsinki and was approved by the Institutional Review Board of Yonsei University Hospital (IRB number: 4-2024-0398). The requirement for informed consent was waived by the Institutional Review Board owing to the retrospective nature of the study, which used de-identified data from the Korean National Health Insurance Service database.

### 2.3. Definition of MASLD

In this study, we used the fatty liver index (FLI) to define SLD. The FLI is calculated using triglyceride levels, gamma-glutamyl transferase (GGT) levels, BMI, and waist circumference (WC); therefore, it is appropriate for identifying hepatic steatosis in large-scale epidemiological studies. Patients with an FLI ≥30 were classified as having SLD, and those with an FLI <30 as non-SLD [22].

Among patients with SLD, CMRFs were assessed [18]. The CMRFs included the following five components: (1) BMI ≥23 kg/m^2^ or WC ≥85 cm (females); (2) fasting glucose ≥100 mg/dL, type 2 diabetes mellitus (DM), or treatment for type 2 DM; (3) blood pressure ≥130/85 mmHg or treatment for hypertension; (4) plasma triglycerides ≥150 mg/dL or lipid-lowering treatment; and (5) plasma high-density lipoprotein cholesterol ≤50 mg/dL (females) or lipid-lowering treatment. For the WC criterion, the cut-off value of 85 cm for females used in this study is widely accepted in South Korea to define central obesity and is considered reflective of morbidity associated with increased waist circumference.

Women with SLD and one or more CMRFs were further classified according to their alcohol intake: those consuming <20 g/day were defined as having MASLD; those consuming 20 to <50 g/day (females) as having MetALD; and those consuming ≥50 g/day as having ALD. However, because patients often underreport their alcohol consumption due to stigma, memory limitations, or cognitive impairment—and because MASLD patients with alcohol use disorders experience poorer outcomes than those without—patients with SLD and ≥1 CMRF who had a diagnosis of alcohol or substance abuse/misuse (Table S1) were classified as having ALD regardless of their reported daily alcohol intake.

### 2.4. Primary Endpoints

The primary endpoints were newly diagnosed CVD, including myocardial infarction (MI) or ischemic stroke. MI was defined as the recording of ICD-10 codes I21 or I22 during hospitalization. Ischemic stroke was defined as the recording of ICD-10 codes I63 or I64 during hospitalization, accompanied by claims for brain magnetic resonance imaging or brain computed tomography. Peripheral arterial events—critical limb ischemia, amputation, and mesenteric ischemia requiring revascularization—were not included in the composite endpoint. Myocardial infarction and ischemic stroke are the outcomes for which claims-based definitions, requiring hospitalization and, for stroke, confirmatory neuroimaging, have been applied consistently in the NHIS database, whereas diagnostic codes alone do not separate acute limb ischemia from chronic peripheral arterial disease and to our knowledge, procedure-based definitions for these events have not been validated in this database. Restricting the endpoint to these two validated outcomes minimized outcome misclassification, at the cost of underestimating the total burden of atherosclerotic disease.

### 2.5. Covariates

Participants’ reproductive and lifestyle factors were assessed using a self-reported questionnaire. Reproductive factors included age at menopause and menarche, parity, breastfeeding status, oral contraceptive use, and menopausal HRT. Smoking status, alcohol use, physical activity, and income level were also assessed and categorized. Anthropometric variables (height, weight, waist circumference, and blood pressure) and laboratory parameters (serum glucose, lipid profile, and creatinine) were measured after an overnight fast. Waist circumference was measured at the midpoint between the lowest rib and the iliac crest. BMI was calculated, with general obesity defined as a BMI ≥25 kg/m^2^; this reflects an Asian-specific threshold, whereas the World Health Organization classifies a BMI of 25.0–29.9 kg/m^2^ as overweight and ≥30 kg/m^2^ as obesity in other populations. Detailed operational definitions of covariates—including categorization of lifestyle factors and income, BMI subgroups, hypertension, dyslipidemia, and chronic kidney disease—are provided in the Supplementary Materials (Supplementary Methods).

### 2.6. Statistical Analysis

The distribution of each continuous variable was examined before analysis using histograms, normal quantile–quantile plots, skewness and kurtosis; formal normality tests were not applied, because at a sample size approaching two million they reject the null hypothesis for departures of no practical consequence. Approximately symmetric variables were presented as means ± standard deviations and compared by independent two-sample t-test. Right-skewed variables—triglycerides, aspartate aminotransferase, alanine aminotransferase, and gamma-glutamyl transferase—were natural-log transformed, presented as geometric means with 95% confidence intervals, and compared on the log scale. Categorical variables were presented as numbers (percentages) and compared using the χ^2^ test. Associations between SLD, obesity type, and subsequent CVD risk were evaluated using multivariable Cox proportional hazards regression models stratified by menopausal status. Adjusted hazard ratios (aHRs) and corresponding 95% confidence intervals (CIs) were calculated after sequential adjustment for demographic, lifestyle, and metabolic covariates.

Three sequential models were fitted. Model 1 was unadjusted, Model 2 was adjusted for age, and Model 3 was additionally adjusted for income, smoking status, cardiometabolic risk factor components, parity, breastfeeding status, and oral contraceptive use (and, in postmenopausal women, hormone replacement therapy and age at menopause). Because several cardiometabolic covariates included in Model 3 may lie on the causal pathway linking obesity, MASLD, and CVD, the less-adjusted models (Models 1 and 2) approximate the total association, whereas Model 3 estimates the association independent of these factors and may underestimate the total effect through overadjustment. Effect modification was assessed using multiplicative interaction terms between MASLD and obesity phenotype.

Associations were additionally expressed on the absolute scale: crude incidence rate differences between obese and non-obese women were calculated within strata of menopausal and MASLD status, with 95% confidence intervals derived from the standard errors of the component rates, and multiplied by 10 to provide an illustrative event difference per 1000 women over 10 years. All statistical analyses were performed using SAS software (version 9.4; SAS Institute, Cary, NC, USA), and a two-sided *p*-value <0.05 was considered statistically significant throughout.

### 2.7. Sensitivity and Subgroup Analyses

Because the fatty liver index and the cardiometabolic risk factor criteria both incorporate body mass index and waist circumference, the definition of MASLD overlaps with the obesity exposures. Because imaging- or histology-based assessment of hepatic steatosis was not available, an anthropometry-independent ascertainment of steatotic liver disease was not possible. We therefore performed an exploratory sensitivity analysis using the triglyceride–glucose (TyG) index as an anthropometry-free metabolic proxy, defined as a TyG index ≥8.5—a threshold proposed for screening for hepatic steatosis rather than for diagnosing it [23]—calculated as ln[fasting triglycerides (mg/dL) × fasting glucose (mg/dL)/2], with at least one cardiometabolic risk factor and alcohol intake <20 g/day. The adiposity criterion (body mass index ≥23 kg/m^2^ or waist circumference ≥85 cm) was removed from both the cardiometabolic risk factor definition and the Model 3 covariate set, so that neither obesity exposure entered the construction of the proxy or the adjustment set.

Among postmenopausal women, associations were additionally re-estimated within strata of age band (50–59, ≥60 years), years since menopause (≤9, ≥10 years; age at the 2009 examination minus self-reported age at menopause), age at menopause (<50, ≥50 years), and hormone replacement therapy use (never, ever), with heterogeneity assessed by multiplicative interaction terms.

## 3. Results

### 3.1. Baseline Characteristics of Study Participants According to Menopausal Status

Table 1 presents a comprehensive comparison of baseline clinical and metabolic characteristics between premenopausal and postmenopausal women. A total of 848,672 premenopausal and 1,110,695 postmenopausal women were included in the study. As expected, postmenopausal women were significantly older (mean age 61.1 ± 8.3 years vs. 44.9 ± 3.8 years, *p* < 0.001) and had a later age at menarche (16.4 ± 1.8 vs. 15.1 ± 1.7 years, *p* < 0.001).

Postmenopausal women had higher BMI and a greater prevalence of MASLD and overall CMRFs compared with premenopausal women (all *p* < 0.001). Geometric mean levels of aspartate aminotransferase, alanine aminotransferase, and GGT were also significantly higher in postmenopausal women. Use of cardiometabolic medications was likewise more common after menopause, with antihypertensive agents prescribed to 33.2% versus 7.0%, lipid-lowering agents to 14.7% versus 2.6%, and glucose-lowering agents to 8.6% versus 1.6% of postmenopausal and premenopausal women, respectively (all *p* < 0.001).

### 3.2. CVD Risk According to MASLD or General/Central Obesity in Premenopausal and Postmenopausal Women

During the follow-up period (median 12.3 years), a total of 106,792 participants (5.45%) developed CVD: 1.24% (n = 10,565) among premenopausal women versus 8.66% (n = 96,227) among postmenopausal women.

**Figure 2:**
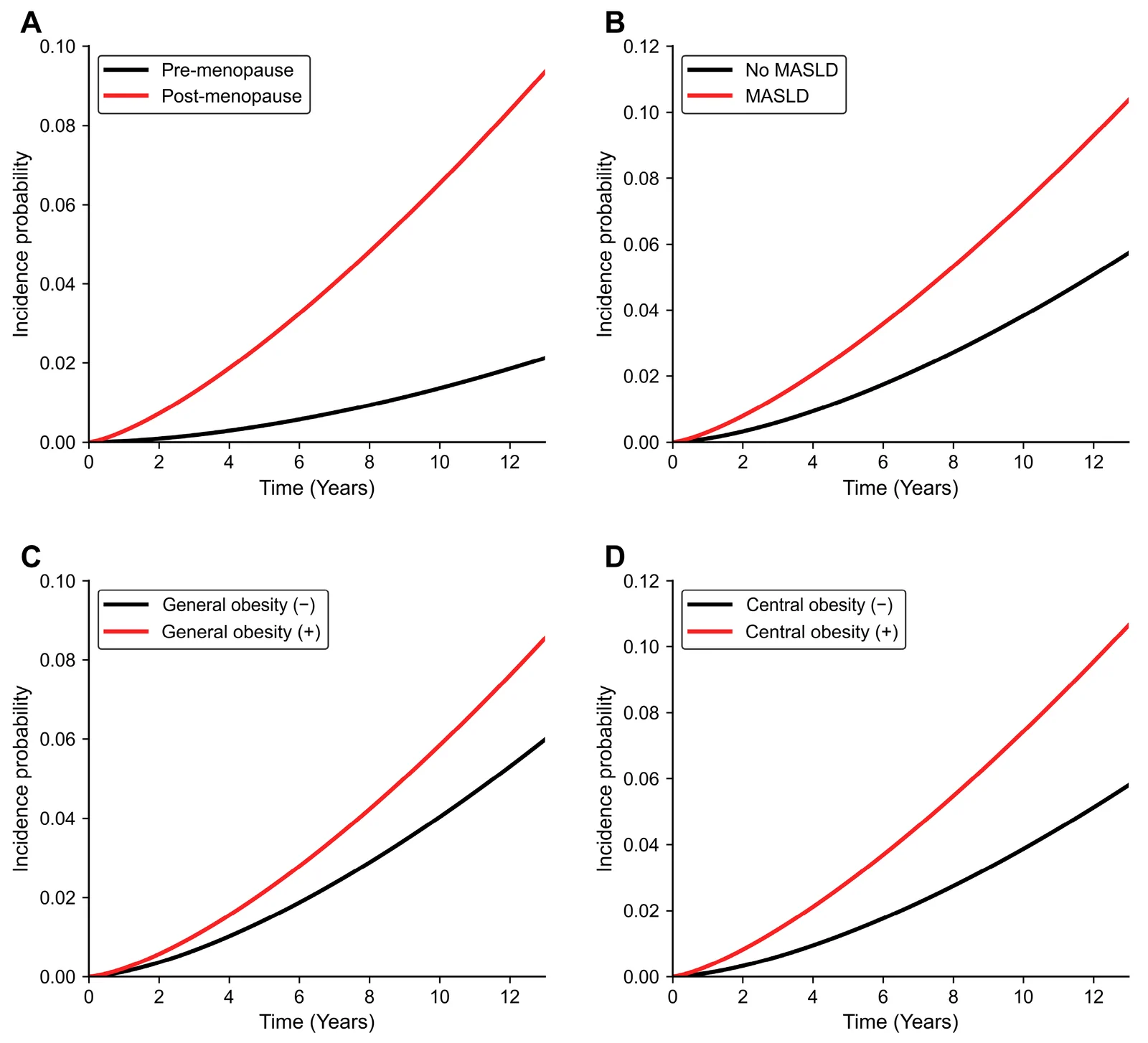
Cumulative incidence plots of cardiovascular disease (CVD): (**A**) Premenopausal vs. postmenopausal women; (**B**) women without metabolic dysfunction-associated steatotic liver disease (MASLD) vs. those with MASLD; (**C**) women without general obesity vs. those with general obesity; (**D**) women without central obesity vs. those with central obesity (all *p* < 0.001 by log-rank test).

Figure 2A shows the CVD risk in premenopausal versus postmenopausal women, with cumulative incidences of 0.42% versus 2.53% at 5 years and 1.36% versus 6.53% at 10 years, respectively (*p* < 0.0001). Figure 2B presents the CVD risk between participants without and with MASLD, with cumulative incidences of 1.32% versus 2.79% at 5 years and 3.83% versus 7.23% at 10 years, respectively (*p* < 0.0001). Figure 2C illustrates the CVD risk in participants without and with general obesity, with cumulative incidences of 1.42% versus 2.14% at 5 years and 4.03% versus 5.84% at 10 years, respectively (*p* < 0.0001). Figure 2D depicts the CVD risk between those without and with central obesity, with cumulative incidences of 1.33% versus 2.87% at 5 years and 3.87% versus 7.43% at 10 years, respectively (*p* < 0.001).

### 3.3. Multivariable Cox Regression Analyses of CVD Risk According to MASLD or General Obesity in Premenopausal and Postmenopausal Women

Table 2 presents the results of multivariable Cox regression analyses stratified by menopausal status, evaluating CVD risk according to MASLD or general obesity. aHRs with 95% CIs were derived from Model 3, which adjusted for age, income, smoking status, cardiometabolic risk factor components, parity, breastfeeding status, and oral contraceptive use in premenopausal women, and for age, income, smoking status, cardiometabolic risk factor components, parity, breastfeeding status, oral contraceptive use, and additionally for hormone replacement therapy and age at menopause in postmenopausal women.

Among participants without MASLD, postmenopausal women were more likely to develop CVD, regardless of general obesity status, than premenopausal women, with incidence rates of 6.56–6.77 versus 1.24–1.47 per 1000 person-years (PYs), respectively. Similarly, among participants with MASLD, postmenopausal women had higher CVD incidence than premenopausal women, regardless of general obesity status, with rates of 9.49–10.06 versus 2.22–2.26 per 1000 PYs, respectively (Table 2).

Among premenopausal women, those with MASLD were more likely to develop CVD than those without MASLD, regardless of general obesity, with incidence rates of 2.22–2.26 versus 1.24–1.47 per 1000 PYs, respectively. Similarly, among postmenopausal women, those with MASLD were more likely to develop CVD than those without MASLD, with rates of 9.49–10.06 versus 6.56–6.77 per 1000 PYs, respectively (Table 2).

Although MASLD was consistently associated with higher CVD risk in both premenopausal and postmenopausal women, Table S2 shows that the magnitude of this association was numerically smaller in women postmenopausal at baseline: from aHRs of 1.378 (without general obesity) and 1.216 (with general obesity) among premenopausal women to 1.164 (without general obesity) and 1.113 (with general obesity) among postmenopausal women.

### 3.4. Multivariable Cox Regression Analyses of CVD Risk According to MASLD or Central Obesity in Premenopausal and Postmenopausal Women

Similarly, Table 3 presents the results of multivariable Cox regression analyses assessing CVD risk according to MASLD status or central obesity in premenopausal and postmenopausal women.

Among participants without MASLD, postmenopausal women were more likely to develop CVD than premenopausal women, regardless of central obesity status, with incidence rates of 6.34–8.88 versus 1.27–1.47 per 1000 PYs, respectively. Similarly, among participants with MASLD, postmenopausal women exhibited higher CVD incidence than premenopausal women, regardless of central obesity status, with incidence rates of 8.28–10.32 versus 2.14–2.29 per 1000 PYs, respectively (Table 3).

Among premenopausal women, those with MASLD had a higher likelihood of developing CVD than those without MASLD, regardless of central obesity status, with incidence rates of 2.14–2.29 versus 1.27–1.47 per 1000 PYs, respectively. Likewise, among postmenopausal women, those with MASLD had higher CVD incidence than those without MASLD, with incidence rates of 8.28–10.32 versus 6.34–8.88 per 1000 PYs, respectively (Table 3).

Although MASLD was consistently associated with higher CVD risk in both premenopausal and postmenopausal women, Table S3 shows that the magnitude of this association was numerically smaller in women postmenopausal at baseline: from aHRs of 1.260 (without central obesity) and 1.265 (with central obesity) among premenopausal women to 1.149 (without central obesity) and 1.120 (with central obesity) among postmenopausal women.

### 3.5. Comparison of General and Central Obesity Associations with CVD Risk

In premenopausal women without MASLD, general obesity was associated with an increased risk of CVD, with an aHR of 1.099 (95% CI, 1.036–1.165), whereas central obesity was not significantly associated with CVD risk (aHR, 1.064; 95% CI, 0.959–1.181). In premenopausal women with MASLD, neither general nor central obesity was associated with CVD risk, with aHRs of 0.970 (95% CI, 0.856–1.098) and 1.069 (95% CI, 0.974–1.172), respectively (Table 2 and Table 3). The MASLD–obesity phenotype interaction was not statistically significant in premenopausal women (*p* for interaction = 0.068 for general obesity and 0.950 for central obesity).

In postmenopausal women without MASLD, general obesity was not associated with CVD risk (aHR, 0.992; 95% CI, 0.971–1.012), whereas central obesity was associated with increased risk (aHR, 1.075; 95% CI, 1.050–1.101). In postmenopausal women with MASLD, the estimate for general obesity was inverse (aHR, 0.948; 95% CI, 0.924–0.973), while central obesity remained positively associated with increased risk (aHR, 1.048; 95% CI, 1.024–1.073) (Table 2 and Table 3). In postmenopausal women, the MASLD–general obesity interaction was statistically significant (*p* for interaction = 0.006), whereas the MASLD–central obesity interaction was not (*p* for interaction = 0.126), indicating that the association of central obesity with CVD risk did not differ significantly by MASLD status.

### 3.6. Absolute Incidence-Rate Differences

Among premenopausal women without MASLD, general obesity corresponded to a crude incidence-rate difference of 0.23 per 1000 person-years (95% CI, 0.14–0.30) (Table S4). Among postmenopausal women, the corresponding difference for central obesity was 2.54 (95% CI, 2.34–2.74) in those without MASLD and 2.03 (95% CI, 1.83–2.24) in those with MASLD. Under a constant-rate assumption these differences correspond illustratively to approximately 2, 25 and 20 additional events per 1000 women over 10 years; they are not adjusted 10-year cumulative-risk differences. The adjusted hazard ratios for general obesity before menopause and for central obesity after menopause were 1.099 and 1.075, with reference incidence rates of 1.24 and 6.34 per 1000 person-years. These absolute estimates are crude and unadjusted.

### 3.7. Exploratory Sensitivity Analysis Using a TyG-Based Metabolic Proxy

Results using the TyG-based metabolic proxy are shown in Table S5. The proxy remained associated with CVD in both menopausal groups, and the association was again smaller in postmenopausal women, although the difference between the menopausal groups was smaller than in the primary analysis and the confidence intervals overlapped (aHR 1.127, 95% CI 1.056–1.202 before versus 1.091, 95% CI 1.070–1.112 after menopause, in women without general obesity). The relative ordering of the two obesity phenotypes was unchanged in every stratum: among postmenopausal women in the proxy-negative group the estimates were 1.120 (95% CI, 1.092–1.149) for central and 1.084 (95% CI, 1.059–1.110) for general obesity, whereas among premenopausal women in the proxy-negative group general obesity was marginally the higher, as in the primary analysis.

Several estimates changed materially under this proxy, and every obesity estimate exceeded 1.0. Most notably, the inverse association between general obesity and CVD among postmenopausal women with MASLD (aHR 0.948) became 1.057 (95% CI, 1.041–1.075) in the corresponding proxy-positive stratum, and the null estimate among postmenopausal women without MASLD (aHR 0.992) became 1.084 (95% CI, 1.059–1.110) in the proxy-negative stratum. Estimates in premenopausal women also increased (general obesity, 0.970 with MASLD to 1.203 in the proxy-positive stratum; central obesity, 1.069 to 1.290), and the liver status × general obesity interaction in postmenopausal women was no longer significant (*p* = 0.0867 for proxy × general obesity, versus 0.0057 for MASLD × general obesity in the primary analysis). A TyG index ≥8.5 was present in 32.9% of premenopausal and 57.6% of postmenopausal women (Table 1); because the TyG index is a marker of insulin resistance rather than a measure of hepatic fat, this proportion is not directly comparable with the prevalence of fatty liver index–based MASLD.

### 3.8. Subgroup Analyses Among Postmenopausal Women

Among postmenopausal women, CVD risk was re-estimated within strata of age band, years since menopause, age at menopause, and hormone replacement therapy use, taking women without steatotic liver disease and without obesity in the same stratum as the reference (Table S6). Estimates for women with MASLD represent the joint association of MASLD and obesity relative to women with neither, rather than the effect of obesity within the MASLD stratum. Associations were largest in the 50–59 year group and in those ≤9 years since menopause, and smaller with older age and longer time since menopause (*p* for interaction <0.0001 for both stratifications and both phenotypes); among women without steatotic liver disease, for example, the aHR for general obesity was 1.089 (95% CI, 1.047–1.133) at 50–59 years and 0.959 (95% CI, 0.937–0.983) at ≥60 years, with the same gradient for central obesity and for women with MASLD. Estimates from the age-band and years-since-menopause stratifications were almost identical. Associations differed modestly by age at menopause for central obesity (*p* = 0.0025) but not for general obesity (*p* = 0.8489), and by hormone replacement therapy use for both phenotypes (*p* = 0.0095 and *p* = 0.0166). General and central obesity were estimated in separate models and were therefore not compared formally; the point estimate for central obesity was numerically higher in all sixteen subgroup-by-liver-status comparisons.

## 4. Discussion

In this large-scale cohort of South Korean women aged ≥40 years, we examined the association of MASLD and obesity type (general vs. central) with CVD risk by menopausal status. Postmenopausal women had a markedly higher CVD incidence than premenopausal women, and MASLD was consistently associated with elevated CVD risk in both groups, although the association was numerically smaller in postmenopausal women. The association of obesity type with CVD risk differed between the two menopausal groups, although the interaction terms tested MASLD × obesity within each group rather than menopause × obesity, so the two groups were not compared formally. In an exploratory sensitivity analysis using a TyG-based metabolic proxy, which contains no anthropometric term, the ordering of the two obesity phenotypes was preserved, but the inverse estimate for general obesity in postmenopausal women with MASLD was not. These findings reinforce the view of MASLD as a systemic, heterogeneous metabolic disorder extending beyond the liver to cardiovascular outcomes.

A recent data-driven cluster analysis identified distinct MASLD subtypes, including a ‘liver-specific’ type with limited cardiovascular risk and a ‘cardiometabolic’ type characterized by dyslipidemia and a high incidence of cardiovascular events [24]. This heterogeneity may underlie our observation that MASLD is associated with CVD risk in both menopausal groups. Meta-analytic data confirm that cardiovascular events are among the most frequent adverse clinical outcomes in this population, exceeding the incidence of hepatic decompensation in most cohorts [25]. Accordingly, joint hepatology and cardiology position statements now recommend that cardiovascular risk be assessed systematically in patients with MASLD [26].

The higher CVD incidence in postmenopausal women is consistent with the known cardiometabolic effects of estrogen deficiency, including increased visceral adiposity, insulin resistance, and chronic inflammation [27]. These changes may contribute to the higher absolute CVD incidence observed in women postmenopausal at baseline [28].

To our knowledge, this is among the largest population-based studies to examine the differential association of MASLD and obesity type with CVD risk in pre- and postmenopausal women within a nationwide East Asian cohort. A plausible mechanism for the positive association observed for central obesity in women postmenopausal at baseline involves redistribution of adipose tissue after estrogen loss: estrogen decline promotes expansion of visceral adipose tissue, a metabolically active endocrine organ that links hormonal changes to vascular injury through pro-inflammatory and pro-thrombotic signaling [29]. The inverse estimate for general obesity among postmenopausal women with MASLD (aHR 0.948) should not be taken as evidence that a higher BMI protects these women. It is more plausibly an instance of the “obesity paradox” [30,31,32], arising within strata themselves defined in part by BMI; the sensitivity analysis was consistent with this interpretation, because the corresponding estimate was 1.057 (95% CI, 1.041–1.075) under the TyG-based metabolic proxy (Table S5); however, that proxy is not a direct measure of hepatic fat and does not establish that definitional overlap was the sole explanation. Consistent with the obesity-paradox interpretation, lean and non-lean populations with MASLD differ in long-term outcomes and in the factors that modify them [33].

These findings may also inform individualized prevention. In premenopausal women without MASLD, weight loss may help reduce CVD risk; once MASLD develops, multimodal approaches—novel pharmacotherapies (e.g., thyroid hormone receptor-β agonists, GLP-1 receptor agonists, SGLT2 inhibitors), diet, and exercise—may also be relevant [34,35]. In selected women with obesity, bariatric intervention produces the largest and most durable histological improvement in steatohepatitis reported to date [36]. For postmenopausal women, targeting central obesity may be particularly relevant, alongside MASLD management and emerging pharmacotherapies that reduce body weight and cardiometabolic risk [37]. Although the adjusted hazard ratios for general obesity before menopause and for central obesity after menopause were of similar magnitude, the absolute excess associated with the latter was roughly ten times larger, reflecting the much higher underlying rate after menopause. The apparent inverse association of general obesity among postmenopausal women with MASLD should be interpreted cautiously, as it may reflect reverse causation, selection bias, collider stratification within MASLD strata, or overadjustment rather than a true effect. This concept is increasingly regarded as an artifact of BMI, which inadequately reflects central adiposity, in both the general population [38] and disease cohorts such as heart failure [39].

The main strengths are scale and exposure detail: nearly two million women followed for a median exceeding 12 years, with anthropometric and laboratory measurements linked to reproductive histories seldom available at this scale. Three features extend the existing evidence: general and central obesity are examined separately rather than as a single exposure; the cohort size allows stable estimation in the four joint MASLD–obesity groups within each menopausal group; and both exposures are already recorded in national health screening, so the proposed stratification requires no additional measurement. Integrating menopausal status and obesity phenotype into public health frameworks—for example, combining the waist circumference already recorded at national screening with routinely collected reproductive histories—offers a low-cost, scalable approach to refining cardiovascular risk stratification. Accordingly, CVD prevention strategies may be tailored to MASLD status and obesity phenotypes. These observations are consistent with the American Heart Association scientific statement, which emphasized abdominal obesity, measured by waist circumference, as a critical risk marker for CVD independent of BMI [40]. Furthermore, sarcopenia should also be considered in CVD prevention strategies [41,42]. Several questions follow from these findings. Prospective studies using imaging-based quantification of hepatic and visceral fat are needed to establish whether these associations persist when steatosis is measured independently of anthropometry [43]. Because fibrosis rather than steatosis governs outcomes in MASLD, adding non-invasive fibrosis assessment would further refine risk stratification—elastography has now been calibrated for this purpose [44], and serum indices such as FIB-4 perform acceptably in high-risk groups [45]. Longitudinal designs following individual women through the menopausal transition would help separate ovarian from chronological aging, and extending the endpoint to peripheral arterial outcomes would capture the atherosclerotic burden omitted here. Finally, whether interventions that preferentially reduce visceral adiposity lower cardiovascular event rates in postmenopausal women with MASLD remains to be tested.

This study has several limitations. Because the FLI and the cardiometabolic criteria both incorporate BMI and waist circumference, the MASLD definition overlaps with the obesity exposures, and Model 3 adjusts for factors that may lie on the causal pathway; an exploratory sensitivity analysis using an anthropometry-free metabolic proxy did not reproduce the inverse estimate for general obesity (Table S5), which is consistent with, but does not prove, bias arising from this overlap. The TyG index is a metabolic proxy rather than a direct measure of hepatic fat, so this sensitivity analysis addresses the anthropometric overlap but does not substitute for imaging- or histology-based ascertainment of steatosis. Age and menopausal status were closely correlated, and age band could not be separated from years since menopause, so the ‘menopausal shift’ should be regarded as hypothesis-generating; natural and surgical menopause were also indistinguishable, since hysterectomy was an exclusion criterion and oophorectomy is not ascertained. Finally, the use of administrative data and surrogate markers, restriction to health-screening participants, and reliance on self-reported menopausal status may have introduced selection, misclassification and residual confounding, and confining the endpoint to myocardial infarction and ischemic stroke is likely to underestimate the total atherosclerotic burden. Menopausal status, obesity and MASLD were ascertained only at the 2009 examination and were not updated during follow-up, so some women classified as premenopausal will have reached menopause during the 12.3 years of observation; all comparisons are therefore between groups defined at baseline rather than within women across the menopausal transition. The interaction terms we report test MASLD × obesity within each menopausal group and not menopause × obesity, so the difference in associations between the two groups is descriptive. Finally, postmenopausal women aged <50 years at the examination were not analysed as a separate stratum in the subgroup analyses.

## 5. Conclusions

In conclusion, MASLD was associated with CVD risk at both menopausal stages, and the associations observed for obesity phenotype differed between them. Among premenopausal women without MASLD, general obesity was associated with increased risk while the estimate for central obesity was directionally similar but not significant; because the confidence intervals overlapped, the two phenotypes cannot be distinguished on the present evidence. Among postmenopausal women, the association with central obesity was the more consistent of the two across strata, corresponding on the crude scale to approximately 20 to 25 additional events per 1000 women over 10 years; because the two phenotypes were modelled separately, this ordering is descriptive; the inverse estimate for general obesity was not reproduced under a TyG-based metabolic proxy that is free of anthropometry. These are cross-group comparisons in a cohort in which menopausal status is closely tied to age, and do not establish that menopause causes a shift in obesity phenotype, that BMI ceases to matter after menopause, or that a higher BMI is protective in MASLD.

## Figures and Tables

**Table 1:** Comparison of clinical characteristics between premenopausal and postmenopausal women.

Variables	Premenopausal (n = 848,672)	Postmenopausal (n = 1,110,695)	*p*-Value
Age, years	44.9 ± 3.8	61.1 ± 8.3	<0.001
Age at menopause, years	-	50.0 ± 3.9	-
Age at menarche, years	15.1 ± 1.7	16.4 ± 1.8	<0.001
Body mass index, kg/m^2^	23.2 ± 3.0	24.1 ± 3.1	<0.001
SLD			<0.001
No SLD	742,465 (87.5)	787,609 (70.9)	
MASLD	106,207 (12.5)	323,086 (29.1)	
Parity			<0.001
None	31,023 (3.7)	19,057 (1.7)	
1	110,978 (13.1)	68,333 (6.2)	
≥2	706,671 (83.3)	1,023,305 (92.1)	
Breastfeeding			<0.001
None	152,952 (18.0)	73,290 (6.6)	
<6 months	209,635 (24.7)	72,504 (6.5)	
6–11 months	224,981 (26.5)	193,215 (17.4)	
≥12 months	261,104 (30.8)	771,686 (69.5)	
Oral contraceptive pill			<0.001
None	740,170 (87.2)	939,019 (84.5)	
<1 year	80,346 (9.5)	104,047 (9.4)	
≥1 year	28,156 (3.3)	67,629 (6.1)	
Hormone replacement therapy			-
None	-	930,774 (83.8)	
<2 years	-	104,753 (9.4)	
2–4 years	-	43,018 (3.9)	
≥5 years	-	32,150 (2.9)	
Low income	210,169 (24.8)	242,540 (21.8)	<0.001
Smoking			<0.001
Never	808,811 (95.3)	1,071,145 (96.4)	
Former	13,212 (1.6)	11,220 (1.0)	
Current	26,649 (3.1)	28,330 (2.6)	
Drinking			<0.001
Non	611,138 (72.0)	973,519 (87.6)	
Mild	221,889 (26.1)	130,024 (11.7)	
Heavy	13,817 (1.6)	6184 (0.6)	
Alcoholic	1828 (0.2)	968 (0.1)	
Regular exercise	146,586 (17.3)	206,602 (18.6)	<0.001
CMRF	622,069 (73.3)	1,006,170 (90.6)	<0.001
General obesity	202,559 (23.9)	402,217 (36.2)	<0.001
Central obesity	93,600 (11.0)	296,652 (26.7)	<0.001
High body mass index or waist circumference	403,516 (47.5)	711,317 (64.0)	<0.001
Hyperglycemia	186,770 (22.0)	395,102 (35.6)	<0.001
High blood pressure	219,695 (25.9)	633,531 (57.0)	<0.001
Hypertriglyceridemia	131,110 (15.4)	423,838 (38.2)	<0.001
Low HDL-C	258,511 (30.5)	515,897 (46.4)	<0.001
TyG ≥8.5	278,830 (32.9)	639,888 (57.6)	<0.001
Medication use			
Antihypertensive	59,450 (7.0)	369,303 (33.2)	<0.001
Lipid-lowering	22,289 (2.6)	163,232 (14.7)	<0.001
Glucose-lowering ^‡^	13,537 (1.6)	95,038 (8.6)	<0.001
Waist circumference, cm	75.0 ± 7.7	79.8 ± 8.2	<0.001
SBP, mmHg	117.0 ± 14.2	125.3 ± 16.1	<0.001
DBP, mmHg	73.0 ± 9.9	76.8 ± 10.1	<0.001
Fasting glucose, mg/dL	93.5 ± 17.5	99.1 ± 23.4	<0.001
Total cholesterol, mg/dL	191.6 ± 33.8	208.8 ± 38.4	<0.001
HDL-C, mg/dL	60.0 ± 30.1	57.7 ± 33.0	<0.001
LDL-C, mg/dL	113.0 ± 35.5	126.3 ± 38.1	<0.001
eGFR, mL/min/1.73 m^2^	89.2 ± 29.1	82.4 ± 28.0	<0.001
TyG	8.3 ± 0.6	8.6 ± 0.6	<0.001
* Triglyceride, mg/dL	87.8 (87.7–87.9)	115.3 (115.1–115.4)	<0.001
* AST, IU/L	20.3 (20.3–20.3)	23.8 (23.7–23.8)	<0.001
* ALT, IU/L	16.5 (16.5–16.5)	20.1 (20.0–20.1)	<0.001
* GGT, IU/L	17.0 (16.9–17.0)	20.3 (20.3–20.4)	<0.001

Values are expressed as n (%) for categorical variables and as means ± standard deviations for approximately symmetrically distributed continuous variables. * Right-skewed variables are presented as geometric means (95% confidence interval) following natural-log transformation; for a log-normal distribution the geometric mean is an estimate of the median. ‡ Oral glucose-lowering agents or insulin. Medication use was ascertained from prescription claims at the time of the 2009 health examination, using the operational definitions given in the Supplementary Methods. Abbreviations: SLD, steatotic liver disease; MASLD, metabolic dysfunction-associated steatotic liver disease; CMRF, cardiometabolic risk factor; HDL-C, high-density lipoprotein cholesterol; SBP, systolic blood pressure; DBP, diastolic blood pressure; LDL-C, low-density lipoprotein cholesterol; eGFR, estimated glomerular filtration rate; AST, aspartate aminotransferase; ALT, alanine aminotransferase; GGT, gamma-glutamyl transferase; TyG, triglyceride–glucose index.

**Table 2:** CVD risk according to MASLD/general obesity status in premenopausal and postmenopausal women.

SLD	Obesity	Incidence Rate *	aHR (95% CI) †			aHR (95% CI) ‡		
			Model 1	Model 2	Model 3	Model 1	Model 2	Model 3
**Premenopausal**								
No SLD	General obesity (−)	1.24	1 (Ref.)	1 (Ref.)	1 (Ref.)	1 (Ref.)	1 (Ref.)	1 (Ref.)
	General obesity (+)	1.47	1.214 (1.147, 1.285)	1.182 (1.117, 1.251)	1.099 (1.036, 1.165)	1.214 (1.147, 1.285)	1.182 (1.117, 1.251)	1.099 (1.036, 1.165)
MASLD	General obesity (−)	2.26	1.925 (1.723, 2.151)	1.831 (1.638, 2.046)	1.378 (1.224, 1.552)	1 (Ref.)	1 (Ref.)	1 (Ref.)
	General obesity (+)	2.22	1.848 (1.751, 1.951)	1.790 (1.696, 1.890)	1.336 (1.236, 1.444)	0.960 (0.853, 1.082)	0.978 (0.868, 1.101)	0.970 (0.856, 1.098)
*p* for interaction						0.0005	0.0048	0.068
**Postmenopausal**								
No SLD	General obesity (−)	6.56	1 (Ref.)	1 (Ref.)	1 (Ref.)	1 (Ref.)	1 (Ref.)	1 (Ref.)
	General obesity (+)	6.77	1.029 (1.009, 1.050)	1.031 (1.011, 1.052)	0.992 (0.971, 1.012)	1.029 (1.009, 1.050)	1.031 (1.011, 1.052)	0.992 (0.971, 1.012)
MASLD	General obesity (−)	10.06	1.542 (1.506, 1.578)	1.328 (1.297, 1.359)	1.164 (1.135, 1.194)	1 (Ref.)	1 (Ref.)	1 (Ref.)
	General obesity (+)	9.49	1.450 (1.429, 1.472)	1.286 (1.267, 1.305)	1.104 (1.082, 1.126)	0.941 (0.918, 0.964)	0.969 (0.945, 0.993)	0.948 (0.924, 0.973)
*p* for interaction						<0.0001	0.0001	0.0057

For premenopausal women: Model 1: Unadjusted, Model 2: Adjusted for age, Model 3: Adjusted for age, income, smoking, cardiometabolic risk factor components, parity, breastfeeding, and oral contraceptives. For postmenopausal women: Model 1: Unadjusted, Model 2: Adjusted for age, Model 3: Adjusted for age, income, smoking, cardiometabolic risk factor components, parity, breastfeeding, oral contraceptives, hormone replacement therapy, and age at menopause. * Per 1000 person-years. † Reference category: no SLD and general obesity (−). ‡ Reference category: general obesity (−) within the same SLD stratum. Abbreviations: CVD, cardiovascular disease; MASLD, metabolic dysfunction-associated steatotic liver disease; SLD, steatotic liver disease; aHR, adjusted hazard ratio; CI, confidence interval.

**Table 3:** CVD risk according to MASLD/central obesity status in premenopausal and postmenopausal women.

SLD	Central Obesity	Incidence Rate *	aHR (95% CI) ^†^			aHR (95% CI) ^‡^		
			Model 1	Model 2	Model 3	Model 1	Model 2	Model 3
**Premenopausal**								
No SLD	Central obesity (−)	1.27	1 (Ref.)	1 (Ref.)	1 (Ref.)	1 (Ref.)	1 (Ref.)	1 (Ref.)
	Central obesity (+)	1.47	1.189 (1.076, 1.313)	1.160 (1.050, 1.282)	1.064 (0.959, 1.181)	1.189 (1.076, 1.313)	1.160 (1.050, 1.282)	1.064 (0.959, 1.181)
MASLD	Central obesity (−)	2.14	1.763 (1.638, 1.896)	1.696 (1.576, 1.824)	1.260 (1.156, 1.374)	1 (Ref.)	1 (Ref.)	1 (Ref.)
	Central obesity (+)	2.29	1.860 (1.749, 1.978)	1.809 (1.701, 1.924)	1.346 (1.243, 1.458)	1.055 (0.964, 1.155)	1.067 (0.975, 1.168)	1.069 (0.974, 1.172)
*p* for interaction						0.0822	0.2224	0.9499
**Postmenopausal**								
No SLD	Central obesity (−)	6.34	1 (Ref.)	1 (Ref.)	1 (Ref.)	1 (Ref.)	1 (Ref.)	1 (Ref.)
	Central obesity (+)	8.88	1.406 (1.374, 1.439)	1.095 (1.070, 1.121)	1.075 (1.050, 1.101)	1.406 (1.374, 1.439)	1.095 (1.070, 1.121)	1.075 (1.050, 1.101)
MASLD	Central obesity (−)	8.28	1.308 (1.281, 1.336)	1.263 (1.237, 1.290)	1.149 (1.122, 1.176)	1 (Ref.)	1 (Ref.)	1 (Ref.)
	Central obesity (+)	10.32	1.637 (1.613, 1.662)	1.322 (1.302, 1.342)	1.204 (1.180, 1.229)	1.252 (1.224, 1.281)	1.047 (1.023, 1.071)	1.048 (1.024, 1.073)
*p* for interaction						<0.0001	0.0062	0.1262

For premenopausal women: Model 1: Unadjusted, Model 2: Adjusted for age, Model 3: Adjusted for age, income, smoking, cardiometabolic risk factor components, parity, breastfeeding, and oral contraceptives. For postmenopausal women: Model 1: Unadjusted, Model 2: Adjusted for age, Model 3: Adjusted for age, income, smoking, cardiometabolic risk factor components, parity, breastfeeding, oral contraceptives, hormone replacement therapy, and age at menopause. * Per 1000 person-years. ^†^ Reference category: no SLD and central obesity (−). ^‡^ Reference category: central obesity (−) within the same SLD stratum. Abbreviations: CVD, cardiovascular disease; MASLD, metabolic dysfunction-associated steatotic liver disease; SLD, steatotic liver disease; aHR, adjusted hazard ratio; CI, confidence interval.

## Data Availability

Restrictions apply to the availability of these data. The data were obtained from the Korean National Health Insurance Service (NHIS) under license for the current study and are available from the corresponding author upon reasonable request and with the permission of the NHIS.

## Supplementary Materials

The following supporting information can be downloaded at https://www.mdpi.com/article/10.3390/biomedicines14091985/s1. Supplementary Methods: Detailed definitions of covariates; Table S1: Definition criteria of concomitant liver disease, alcohol-associated liver disease, or alcohol abuse/misuse; Table S2: CVD risk according to general obesity/MASLD status in premenopausal and postmenopausal women; Table S3: CVD risk according to central obesity/MASLD status in premenopausal and postmenopausal women; Table S4: Crude incidence-rate differences and illustrative 10-year event differences for MASLD and obesity phenotype; Table S5: CVD risk according to obesity and TyG-based metabolic proxy status in premenopausal and postmenopausal women; Table S6: CVD risk according to obesity phenotype among postmenopausal women, stratified by age, time since menopause, age at menopause, and hormone replacement therapy use.

## Abbreviations

The following abbreviations are used in this manuscript:

aHR	adjusted hazard ratio
ALD	alcohol-associated liver disease
ALT	alanine aminotransferase
AST	aspartate aminotransferase
BMI	body mass index
CI	confidence interval
CMRF	cardiometabolic risk factor
CVD	cardiovascular disease
DM	diabetes mellitus
FLI	fatty liver index
GGT	gamma-glutamyl transferase
HRT	hormone replacement therapy
ICD-10	International Statistical Classification of Diseases and Related Health Problems, Tenth Revision
MASLD	metabolic dysfunction-associated steatotic liver disease
MetALD	metabolic dysfunction-associated steatotic liver disease with increased alcohol intake
MI	myocardial infarction
NHIS	National Health Insurance Service
PY	person-year
SLD	steatotic liver disease
TyG	triglyceride–glucose index
WC	waist circumference
