# Supplementary material for: Associations of General and Central Obesity with Cardiovascular Risk by Menopausal and MASLD Status
Source: Biomedicines. 2026 Sep 3;14(9):1985. doi: 10.3390/biomedicines14091985 (PMC13604105; doi:10.3390/biomedicines14091985)
Supplement: Supplementary file 1 [file biomedicines-14-01985-s001.zip › biomedicines-4503979-supplementary.pdf]

Associations of General and Central Obesity with Cardiovascular Risk by Menopausal and MASLD Status

Dong Yun Kim [1,2,3,†], Kyu-Na Lee [4,†], Kyung-Do Han [5,†] and Beom Kyung Kim [1,2,3,*]

[1] Department of Internal Medicine, Yonsei University College of Medicine, 50-1 Yonsei-ro, Seodaemun-gu, Seoul 03722, Republic of Korea

[2] Institute of Gastroenterology, Yonsei University College of Medicine, 50-1 Yonsei-ro, Seodaemun-gu, Seoul 03722, Republic of Korea

[3] Yonsei Liver Center, Severance Hospital, Yonsei University Health System, 50-1 Yonsei-ro, Seodaemun-gu, Seoul 03722, Republic of Korea

[4] Department of Public Health, The Catholic University of Korea, 222 Banpo-daero, Seocho-gu, Seoul 06591, Republic of Korea

[5] Department of Statistics and Actuarial Science, Soongsil University, 369 Sangdo-ro, Dongjak-gu, Seoul 06978, Republic of Korea

* Correspondence: beomkkim@yuhs.ac

† These authors contributed equally to this work.

Supplementary Materials

Supplementary Methods. Detailed definitions of covariates.

Smoking status was classified as never, former, or current smoker. Alcohol use was categorized as non-drinker, mild drinker, heavy drinker, or alcoholic. Physical activity was defined as engaging in moderate-intensity exercise ≥5 days per week or vigorous exercise ≥3 days per week. Low income was defined as belonging to the lowest income quartile or receiving medical aid based on monthly health insurance premiums. Body mass index (BMI) was categorized into five groups: <18.5, 18.5–22.9, 23.0–24.9, 25.0–29.9, and ≥30.0 kg/m². Hypertension was defined as a systolic or diastolic blood pressure ≥140/90 mmHg or a claim for antihypertensive medication under ICD-10 codes I10–I13 and I15. Dyslipidemia was defined as a serum total cholesterol level ≥240 mg/dL (to convert cholesterol to mmol/L, multiply by 0.0259) or lipid-lowering medication claims under ICD-10 code E78. Chronic kidney disease was identified as an estimated glomerular filtration rate <60 mL/min/1.73 m², calculated using the Modification of Diet in Renal Disease equation. Medication use reported in Table 1 was ascertained from prescription claims at the time of the 2009 health examination: antihypertensive agents were identified by claims for antihypertensive medication under ICD-10 codes I10–I13 and I15, lipid-lowering agents by claims under ICD-10 code E78, and glucose-lowering agents as oral glucose-lowering agents or insulin.

Table S1. Definition criteria of concomitant liver disease, alcohol-associated liver disease, or alcohol abuse/misuse.

Disease	ICD-10
Alcohol-associated liver disease or alcohol abuse/misuse	E24.4, F10, G31.2, G62.1, G72.1, I42.6, K29.2, K70, K86.0, O35.4, R78.0, T51.0, T51.8, T51.9, X65, Y15, Y57.3, Y90, Y91, Z50.2, Z71.4
Concomitant liver disease	
Drug-induced liver disease	K71
Viral hepatitis (e.g. hepatitis B, C)	B15-19
Hepatic veno-occlusive disease (Budd-Chiari)	I82
Liver abscess	K75.0, A06.4
Hemochromatosis	E83.1
Wilson's disease	E83.0
Alpha-1 antitrypsin deficiency	E88.0
Autoimmune hepatitis	K75.4
Primary biliary cholangitis	K74.3, K74.4
Other cholangitis	K83
Glycogen storage disease	E74

Table S2. CVD risk according to general obesity/MASLD status in premenopausal and postmenopausal women.

Obesity	SLD	Incidence rate*	aHR (95% CI) †			aHR (95% CI) ‡		
			Model 1	Model 2	Model 3	Model 1	Model 2	Model 3
**Premenopausal**								
General obesity (-)	No SLD	1.24	1 (Ref.)	1 (Ref.)	1 (Ref.)	1 (Ref.)	1 (Ref.)	1 (Ref.)
	MASLD	2.26	1.925 (1.723, 2.151)	1.831 (1.638, 2.046)	1.378 (1.224, 1.552)	1.925 (1.723, 2.151)	1.831 (1.638, 2.046)	1.378 (1.224, 1.552)
General obesity (+)	No SLD	1.47	1.214 (1.147, 1.285)	1.182 (1.117, 1.251)	1.099 (1.036, 1.165)	1 (Ref.)	1 (Ref.)	1 (Ref.)
	MASLD	2.22	1.848 (1.751, 1.951)	1.790 (1.696, 1.890)	1.336 (1.236, 1.444)	1.523 (1.418, 1.634)	1.515 (1.411, 1.626)	1.216 (1.119, 1.322)
P for interaction						0.0005	0.0048	0.068
**Postmenopausal**								
General obesity (-)	No SLD	6.56	1 (Ref.)	1 (Ref.)	1 (Ref.)	1 (Ref.)	1 (Ref.)	1 (Ref.)
	MASLD	10.06	1.542 (1.506, 1.578)	1.328 (1.297, 1.359)	1.164 (1.135, 1.194)	1.542 (1.506, 1.578)	1.328 (1.297, 1.359)	1.164 (1.135, 1.194)
General obesity (+)	No SLD	6.77	1.029 (1.009, 1.050)	1.031 (1.011, 1.052)	0.992 (0.971, 1.012)	1 (Ref.)	1 (Ref.)	1 (Ref.)
	MASLD	9.49	1.450 (1.429, 1.472)	1.286 (1.267, 1.305)	1.104 (1.082, 1.126)	1.409 (1.379, 1.439)	1.247 (1.221, 1.274)	1.113 (1.087, 1.140)
P for interaction						<0.0001	0.0001	0.0057

For premenopausal women:
Model 1: Unadjusted
Model 2: Adjusted for age
Model 3: Adjusted for age, income, smoking, cardiometabolic risk factor components, parity, breastfeeding, and oral contraceptives
For postmenopausal women:
Model 1: Unadjusted
Model 2: Adjusted for age
Model 3: Adjusted for age, income, smoking, cardiometabolic risk factor components, parity, breastfeeding, oral contraceptives, hormone replacement therapy, and age at menopause
* Per 1000 person-years.
† Reference category: no SLD and general obesity (-). ‡ Reference category: no SLD within the same general obesity stratum.
Abbreviations: CVD, cardiovascular disease; MASLD, metabolic dysfunction-associated steatotic liver disease; SLD, steatotic liver disease; aHR, adjusted hazard ratio; CI, confidence interval

Table S3. CVD risk according to central obesity/MASLD status in premenopausal and postmenopausal women.

Central Obesity	SLD	Incidence rate*	aHR (95% CI) †			aHR (95% CI) ‡		
			Model 1	Model 2	Model 3	Model 1	Model 2	Model 3
**Premenopausal**								
Central Obesity (-)	No SLD	1.27	1 (Ref.)	1 (Ref.)	1 (Ref.)	1 (Ref.)	1 (Ref.)	1 (Ref.)
	MASLD	2.14	1.763 (1.638, 1.896)	1.696 (1.576, 1.824)	1.260 (1.156, 1.374)	1.763 (1.638, 1.896)	1.696 (1.576, 1.824)	1.260 (1.156, 1.374)
Central Obesity (+)	No SLD	1.47	1.189 (1.076, 1.313)	1.160 (1.050, 1.282)	1.064 (0.959, 1.181)	1 (Ref.)	1 (Ref.)	1 (Ref.)
	MASLD	2.29	1.860 (1.749, 1.978)	1.809 (1.701, 1.924)	1.346 (1.243, 1.458)	1.565 (1.398, 1.751)	1.559 (1.393, 1.746)	1.265 (1.126, 1.422)
P for interaction						0.0822	0.2224	0.9499
**Postmenopausal**								
Central Obesity (-)	No SLD	6.34	1 (Ref.)	1 (Ref.)	1 (Ref.)	1 (Ref.)	1 (Ref.)	1 (Ref.)
	MASLD	8.28	1.308 (1.281, 1.336)	1.263 (1.237, 1.290)	1.149 (1.122, 1.176)	1.308 (1.281, 1.336)	1.263 (1.237, 1.290)	1.149 (1.122, 1.176)
Central Obesity (+)	No SLD	8.88	1.406 (1.374, 1.439)	1.095 (1.070, 1.121)	1.075 (1.050, 1.101)	1 (Ref.)	1 (Ref.)	1 (Ref.)
	MASLD	10.32	1.637 (1.613, 1.662)	1.322 (1.302, 1.342)	1.204 (1.180, 1.229)	1.164 (1.136, 1.193)	1.207 (1.177, 1.238)	1.120 (1.094, 1.147)
P for interaction						<0.0001	0.0062	0.1262

For premenopausal women:
Model 1: Unadjusted
Model 2: Adjusted for age
Model 3: Adjusted for age, income, smoking, cardiometabolic risk factor components, parity, breastfeeding, and oral contraceptives
For postmenopausal women:
Model 1: Unadjusted
Model 2: Adjusted for age
Model 3: Adjusted for age, income, smoking, cardiometabolic risk factor components, parity, breastfeeding, oral contraceptives, hormone replacement therapy, and age at menopause
* Per 1000 person-years.
† Reference category: no SLD and central obesity (-). ‡ Reference category: no SLD within the same central obesity stratum.
Abbreviations: CVD, cardiovascular disease; MASLD, metabolic dysfunction-associated steatotic liver disease; SLD, steatotic liver disease; aHR, adjusted hazard ratio; CI, confidence interval

Table S4. Crude incidence-rate differences and illustrative 10-year event differences for MASLD and obesity phenotype.

SLD	Incidence rate (95% CI) *		Incidence rate difference (95% CI) †	Illustrative event difference per 1,000 women over 10 years ‡	aHR (95% CI) §
	Obesity (-)	Obesity (+)			
**Premenopausal, general obesity**					
No SLD	1.24 (1.22, 1.27)	1.47 (1.39, 1.55)	0.23 (0.14, 0.30)	2.3	1.099 (1.036, 1.165)
MASLD	2.26 (2.03, 2.52)	2.22 (2.12, 2.33)	-0.04 (-0.31, 0.22)	-0.4	0.970 (0.856, 1.098)
**Postmenopausal, general obesity**					
No SLD	6.56 (6.51, 6.62)	6.77 (6.65, 6.89)	0.21 (0.07, 0.34)	2.1	0.992 (0.971, 1.012)
MASLD	10.06 (9.85, 10.29)	9.49 (9.38, 9.61)	-0.57 (-0.82, -0.33)	-5.7	0.948 (0.924, 0.973)
**Premenopausal, central obesity**					
No SLD	1.27 (1.24, 1.30)	1.47 (1.33, 1.62)	0.20 (0.06, 0.35)	2.0	1.064 (0.959, 1.181)
MASLD	2.14 (2.01, 2.31)	2.29 (2.16, 2.42)	0.13 (-0.07, 0.33)	1.3	1.069 (0.974, 1.172)
**Postmenopausal, central obesity**					
No SLD	6.34 (6.29, 6.40)	8.88 (8.69, 9.07)	2.54 (2.34, 2.74)	25.4	1.075 (1.050, 1.101)
MASLD	8.28 (8.13, 8.45)	10.32 (10.19, 10.45)	2.03 (1.83, 2.24)	20.3	1.048 (1.024, 1.073)

General obesity was defined as a body mass index ≥25 kg/m² and central obesity as a waist circumference ≥85 cm.

* Crude incidence rate per 1,000 person-years; point estimates are reproduced from Tables 2 and 3, and the confidence intervals were derived from the underlying event counts and person-years.

† Incidence rate difference, obesity (+) minus obesity (-), calculated from the underlying event counts and person-years within each stratum of menopausal and MASLD status. Because the incidence-rate point estimates displayed here were reproduced from Tables 2 and 3, the rate difference need not equal the arithmetic difference between the displayed point estimates.

‡ Multiplying the crude incidence-rate difference by 10 provides an illustrative estimate under a constant-rate assumption; it is not an adjusted 10-year cumulative-risk difference, and it is not corrected for competing risks.

§ Adjusted hazard ratio for obesity (+) versus obesity (-) within the same MASLD stratum, reproduced from Model 3 of Tables 2 and 3.

Abbreviations: SLD, steatotic liver disease; MASLD, metabolic dysfunction-associated steatotic liver disease; CI, confidence interval; aHR, adjusted hazard ratio

Table S5. CVD risk according to obesity and TyG-based metabolic proxy status in premenopausal and postmenopausal women.

TyG-based proxy status	Obesity	General obesity		Central obesity	
		aHR (95% CI) †	aHR (95% CI) ‡	aHR (95% CI) †	aHR (95% CI) ‡
**Premenopausal**					
Proxy-negative	Obesity (-)	1 (Ref.)	1 (Ref.)	1 (Ref.)	1 (Ref.)
Proxy-negative	Obesity (+)	1.194 (1.123, 1.271)	1.194 (1.123, 1.271)	1.179 (1.079, 1.289)	1.179 (1.079, 1.289)
Proxy-positive	Obesity (-)	1.127 (1.056, 1.202)	1 (Ref.)	1.121 (1.055, 1.191)	1 (Ref.)
Proxy-positive	Obesity (+)	1.356 (1.261, 1.458)	1.203 (1.126, 1.286)	1.446 (1.331, 1.572)	1.290 (1.198, 1.390)
P for interaction		0.8677		0.1257	
**Postmenopausal**					
Proxy-negative	Obesity (-)	1 (Ref.)	1 (Ref.)	1 (Ref.)	1 (Ref.)
Proxy-negative	Obesity (+)	1.084 (1.059, 1.110)	1.084 (1.059, 1.110)	1.120 (1.092, 1.149)	1.120 (1.092, 1.149)
Proxy-positive	Obesity (-)	1.091 (1.070, 1.112)	1 (Ref.)	1.080 (1.060, 1.100)	1 (Ref.)
Proxy-positive	Obesity (+)	1.154 (1.130, 1.177)	1.057 (1.041, 1.075)	1.202 (1.178, 1.227)	1.114 (1.096, 1.132)
P for interaction		0.0867		0.6958	

A TyG-based metabolic proxy for steatotic liver disease was defined as a triglyceride–glucose index ≥8.5, calculated as ln[fasting triglycerides (mg/dL) × fasting glucose (mg/dL)/2], together with at least one cardiometabolic risk factor and alcohol intake <20 g/day. The TyG index is a marker of insulin resistance and not a direct measure of hepatic fat, so this proxy is not an independent definition of steatotic liver disease. The adiposity criterion (body mass index ≥23 kg/m² or waist circumference ≥85 cm) was excluded both from the cardiometabolic risk factor definition and from the covariate set, so that neither obesity exposure entered the construction of the proxy or the adjustment set. Proxy-positive denotes women meeting this proxy and proxy-negative those not meeting it.

In the left column group obesity denotes general obesity (body mass index ≥25 kg/m²); in the right column group it denotes central obesity (waist circumference ≥85 cm).

All estimates are from Model 3: adjusted for age, income, smoking, the four non-adiposity cardiometabolic risk factor components, parity, breastfeeding, and oral contraceptives; and additionally, in postmenopausal women, for hormone replacement therapy and age at menopause.

† Reference category: proxy-negative and obesity (-).

‡ Reference category: obesity (-) within the same proxy stratum.

P for interaction is the P value of the multiplicative proxy × obesity term.

Abbreviations: CVD, cardiovascular disease; TyG, triglyceride–glucose index; aHR, adjusted hazard ratio; CI, confidence interval

Table S6. CVD risk according to obesity phenotype among postmenopausal women, stratified by age, time since menopause, age at menopause, and hormone replacement therapy use.

Subgroup	SLD	General obesity aHR (95% CI) *	Central obesity aHR (95% CI) *
**Age band**			
50–59 years	No SLD	1.089 (1.047, 1.133)	1.159 (1.094, 1.226)
50–59 years	MASLD	1.225 (1.183, 1.268)	1.324 (1.276, 1.374)
≥60 years	No SLD	0.959 (0.937, 0.983)	1.056 (1.029, 1.084)
≥60 years	MASLD	1.072 (1.049, 1.096)	1.180 (1.155, 1.205)
P for interaction		<0.0001	<0.0001
**Years since menopause †**			
≤9 years	No SLD	1.087 (1.048, 1.127)	1.163 (1.106, 1.223)
≤9 years	MASLD	1.221 (1.182, 1.260)	1.320 (1.276, 1.365)
≥10 years	No SLD	0.951 (0.928, 0.975)	1.049 (1.022, 1.078)
≥10 years	MASLD	1.064 (1.041, 1.088)	1.172 (1.147, 1.198)
P for interaction		<0.0001	<0.0001
**Age at menopause**			
<50 years	No SLD	0.984 (0.951, 1.018)	1.036 (0.997, 1.077)
<50 years	MASLD	1.102 (1.072, 1.133)	1.185 (1.153, 1.219)
≥50 years	No SLD	0.998 (0.973, 1.023)	1.100 (1.068, 1.133)
≥50 years	MASLD	1.109 (1.084, 1.135)	1.218 (1.190, 1.247)
P for interaction		0.8489	0.0025
**Hormone replacement therapy**			
Never used	No SLD	0.980 (0.959, 1.002)	1.062 (1.036, 1.089)
Never used	MASLD	1.101 (1.078, 1.124)	1.203 (1.179, 1.229)
Ever used	No SLD	1.076 (1.019, 1.136)	1.196 (1.150, 1.282)
Ever used	MASLD	1.147 (1.093, 1.203)	1.225 (1.165, 1.288)
P for interaction		0.0095	0.0166

All analyses were restricted to postmenopausal women. General obesity was defined as a body mass index ≥25 kg/m² and central obesity as a waist circumference ≥85 cm.

* Adjusted hazard ratio for women with obesity relative to women without steatotic liver disease and without obesity in the same subgroup. For the "No SLD" rows this estimates the association of obesity alone; for the "MASLD" rows it estimates the joint association of MASLD and obesity. Estimates are from Model 3, with the covariate set of Tables 2 and 3, fitted within each subgroup.

P for interaction is the P value of the multiplicative obesity × stratifying variable term. Postmenopausal women aged <50 years at the 2009 examination were not analysed as a separate stratum.

† Years since menopause was calculated as age at the 2009 health examination minus self-reported age at menopause.

Abbreviations: CVD, cardiovascular disease; SLD, steatotic liver disease; MASLD, metabolic dysfunction-associated steatotic liver disease; aHR, adjusted hazard ratio; CI, confidence interval
